# Nitromusk and Polycyclic Musk Compounds as Long-Term Inhibitors of Cellular Xenobiotic Defense Systems Mediated by Multidrug Transporters

**DOI:** 10.1289/ehp.7301

**Published:** 2004-09-30

**Authors:** Till Luckenbach, David Epel

**Affiliations:** Hopkins Marine Station of Stanford University, Pacific Grove, California, USA

**Keywords:** chemosensitizers, fragrances, MDR, multidrug resistance, multixenobiotic resistance, MXR, *Mytilus californianus*, nitromusks, polycyclic musks

## Abstract

Synthetic musk compounds, widely used as fragrances in consumer products, have been detected in human tissue and, surprisingly, in aquatic organisms such as fish and mollusks. Although their persistence and potential to bioaccumulate are of concern, the toxicity and environmental risks of these chemicals are generally regarded as low. Here, however, we show that nitromusks and polycyclic musks inhibit the activity of multidrug efflux transporters responsible for multixenobiotic resistance (MXR) in gills of the marine mussel *Mytilus californianus*. The IC_10_ (concentration that inhibits 10%) values for the different classes of musks were in the range of 0.09–0.39 μM, and IC_50_ values were 0.74–2.56 μM. The immediate consequence of inhibition of efflux transporters is that normally excluded xenobiotics will now be able to enter the cell. Remarkably, the inhibitory effects of a brief 2-hr exposure to musks were only partially reversed after a 24- to 48-hr recovery period in clean seawater. This unexpected consequence of synthetic musks—a long-term loss of efflux transport activity—will result in continued accumulation of normally excluded toxicants even after direct exposure to the musk has ended. These findings also point to the need to determine whether other environmental chemicals have similar long-term effects on these transporters. The results are relevant to human health because they raise the possibility that exposure to common xenobiotics and pharmaceuticals could cause similar long-term inhibition of these transporters and lead to increased exposure to normally excluded toxicants.

Artificial musk compounds are widely used as inexpensive fragrances and fixatives in personal care products, including detergents, cleaning agents, air fresheners, and cosmetic products (names and structures are shown in Table 1). The worldwide production of musks increased from about 7,000 to 8,000 metric tons/year between 1987 and 1996, with a concurrent production shift from nitromusks to polycyclic musks (Rimkus 1999). The most widely used polycyclic musk is Galaxolide (HHCB), followed by Tonalide (AHTN) (Rimkus 1999), and production of these two polycyclics was about 1,800 metric tons in 2000 in Europe, whereas production of other polycyclic musks was < 20 metric tons (Kupper et al. 2004). Musk xylene (MX) is the most common nitromusk, but use was discontinued in Japan and a voluntary ban is in force in Germany (Käfferlein et al. 1998). Use is still high in the United States, although it is banned in products with a risk of oral uptake (e.g., lipsticks). Toxicologic data have not suggested severe health risks associated with artificial musks, although long-term carcinogenic effects cannot be ruled out (Api et al. 2004; Frosch et al. 1995; Käfferlein and Angerer 2001; Schmeiser et al. 2001); there is also concern about accumulation in adipose tissue, blood plasma, and breast milk (Käfferlein and Angerer 2001; Liebl and Ehrenstorfer 1993; Ott et al. 1999; Rimkus and Wolf 1996).

More than two decades ago Yamagishi et al. (1981, 1983) reported the presence of musks in the aquatic environment and biota in Japan, leading to similar investigations in Europe and North America (Balk and Ford 1999a; Bester et al. 1998; Draisci et al. 1998; Gatermann et al. 2002; Käfferlein et al. 1998; Kallenborn et al. 1999; Osemwengi and Steinberg 2001; Paasivirta et al. 2002; Peck and Hornbuckle 2004; Ricking et al. 2003; Rimkus 1999; Rimkus et al. 1997, 1999; Rimkus and Wolf 1995; Tas and Balk 1997; Tas et al. 1997). These studies show that synthetic musks are widespread in marine and freshwater environments and bioaccumulate to a high degree in fish and invertebrates. Because acute and chronic toxicity thresholds for musks in invertebrate and fish species are much higher than the environmentally measured levels (Balk and Ford 1999b; Behechti et al. 1998; Breitholtz et al. 2003; Chou and Dietrich 1999b; Wollenberger et al. 2003), the environmental risks posed by musks are assumed to be low (Balk et al. 2001). Musks show low binding affinity to estrogen receptors, and their environmental impact as endocrine disruptors through this pathway is also regarded as low (Bitsch et al. 2002; Chou and Dietrich 1999a), although indirect endocrine effects such as inhibition of hormone synthesis (Kester et al. 2000; Sanderson et al. 2001) have not been examined.

However, it has been proposed that pharmaceutical and personal care products (PPCPs), a vast group of environmental contaminants including the artificial musks, might affect organisms through interference with multidrug/multixenobiotic resistance (MDR/MXR) efflux transporters (Daughton and Ternes 1999; Epel and Smital 2001; Smital et al. 2004). The activity of these transporters provides a first line of defense to prevent accumulation of xenobiotics in cells. Inhibition of this cellular defense mechanism increases the sensitivity of cells to xenobiotics by permitting normally excluded toxicants to enter the cell (Epel 1998; Kurelec 1992).

The transporter proteins responsible for MXR include P-glycoprotein (P-gp), multi-drug-resistance protein (MRP), and other members of the ABC (ATP-binding cassette) family of transport proteins. A characteristic feature of these efflux transporters is affinity for a diverse array of substrates. For instance, P-gp acts on a large number of chemically unrelated substrates whose common properties are small size, moderate hydrophobicity, and positively charged domains (Bain et al. 1997). Although this low specificity may provide an advantage by enabling the system to cope with “new” chemicals (e.g., environmental pollutants), a disadvantage is that the transporter capacity is more easily saturated in the presence of many substrates, and its protective role can then be lost. This subversion of the MXR defense by multiple substrates or inhibitors of efflux transporters is referred to as chemosensitization, and compounds that cause this behavior are referred to as chemosensitizers (Epel 1998; Kurelec 1997; Smital and Kurelec 1998b). We hypothesized that the synthetic musks, which are small-molecular-weight, moderately hydrophobic compounds, might be such chemosensitizers, and preliminary studies indeed showed inhibition of efflux transporters in gill tissues of a marine mussel (Luckenbach et al. 2004).

In this article we describe the inhibitory potencies of six artificial musk compounds and report the remarkable finding of continued inhibition of transport activity for 24–48 hr after a short (2 hr) exposure to musks. Although of low toxicity themselves, musks could therefore enhance toxicity of other compounds by blocking the MXR defense system. These results strongly affirm the hypothesis of Kurelec and collaborators, that indirect effects of environmental chemicals as efflux transporter chemosensitizers could be of major importance (Epel 1998; Kurelec 1995, 1997; Smital and Kurelec 1998b), but, additionally, our results indicate that the effects of chemosensitizers might continue long after the exposure event.

## Chemicals.

Musk ketone (MK), MX, HHCB (1,3,4,6,7,8-hexahydro-4,6,6,7,8,8-hexamethyl-cyclopenta-γ-[2]-benzopyran), and Celestolide (ADBI; 4-acetyl-1,1-dimethyl-6-*tert*-butylindane) were gifts from International Flavors & Fragrances Inc. (IFF; Union Beach, NJ); AHTN (7-acetyl-1,1,3,4,4,6-hexamethyl-1,2,3,4-tetrahydronaphthalene) was obtained from Bush Boake Allen Inc. (Jacksonville, FL); and Traseolide (ATII; 5-acetyl-1,1,2,6-tetramethyl-3-isopropylindane) was obtained from Quest International (Mount Olive, NJ). Rhodamine B, (±)-verapamil hydrochloride, and quinidine were purchased from Sigma Chemical Company (St. Louis, MO). Purity of MK, MX, verapamil, and quinidine was ≥ 99%. Purity data were not available for the other musk compounds used. Stocks of rhodamine B in ultrapure water and ethanol stocks of the musks, verapamil, and quinidine were stored in glass flasks at 4°C in the dark.

### Animals and tissue preparation.

California mussels (*Mytilus californianus* Conrad, 1837), with valve lengths ranging from 70 to 95 mm, were collected from the rocky intertidal zone at Hopkins Marine Station (Pacific Grove, CA) and maintained in tanks with running seawater (approximately 15°C) for at least 24 hr and no longer than 2 weeks before experiments. Experiments were performed with gill tissue, which shows high efflux transporter activity (Cornwall et al. 1995).

The mussels were opened by cutting through the adductor muscles with a sharp knife, and the gills were excised with fine scissors and placed in filtered seawater (FSW; 15°C). Each gill consists of two tissue lobes, which were separated at their dorsal connection. To obtain tissue pieces of equal size, dermatology biopsy punches (Acuderm, Fort Lauderdale, FL) were used to excise disks (diameter, 5 mm) from the tissue lobes. The tissue disks that result from this procedure consist of a double layer of lamellae (descending and ascending arm) that are connected by interlamellar tissue bridges. Approximately 55–70 disks per individual were obtained from mussels of the size used for these experiments. Mucus was removed from the gills with forceps before preparing the disks; the tissue disks were flushed and kept in FSW until use.

The sex of each animal was determined from visual inspection of gametes, which are clearly distinguishable by shape (White 1937). A piece of gonad tissue was dipped into a droplet of seawater on a microscope slide, covered with a cover slip, and examined under a light microscope equipped with phase contrast at 400×.

### Competitive substrate/transporter inhibition assay and long-term inhibition.

The fluorescent dye rhodamine B was used as an indicator of efflux transporter activity. Inhibition of transporter activity by a test compound is indicated by increased fluorescence due to higher accumulation of rhodamine B in the cell (Neyfakh 1988). Test solutions were prepared in FSW with 1 μM rhodamine B. Stocks of the test compounds dissolved in ethanol were added to FSW to the desired concentrations, and all solutions were adjusted to 1% ethanol along with an ethanol control. Separate experiments indicated that this ethanol concentration had no effect on transport activity (data not shown). Incubations were performed in glass petri dishes (diameter, 5 cm) in a volume of 10 mL.

Gill tissue disks were incubated in test solutions with gentle rocking for 90 min at 15°C in the dark (*n* = 2–5 tissue disks per dish). After exposure, the tissue disks were briefly washed twice in FSW to remove external rhodamine B, frozen on dry ice or in liquid nitrogen, and stored at −20°C in the dark. For analysis, rhodamine B was extracted by sonicating the tissue in 200 μL *n*-butanol until fully homogenized. *n*-Butanol was used for extractions because, of the several extraction media tested (water, phosphate-buffered saline, some organic solvents), fluorescence signals from rhodamine B were strongest with this solvent. The sonicates were then centrifuged at 13,000*g* for 5 min, and the supernatant was transferred to black 96-well microplates (Packard Instrument Co., Meriden, CT). The amount of dye in the supernatant was determined with a fluorescence microplate reader (Packard FluoroCount; emission, 540 nm; excitation, 580 nm). Rhodamine B extracts were kept dark and on ice at all times. At each reading, background fluorescence was determined with pure *n*-butanol and subtracted from each value. Background fluorescence values of gill tissue with no rhodamine added were similar to that of the *n*-butanol extraction medium.

To examine the duration of inhibitory effects after exposure to test compounds, six to nine gill tissue disks were incubated with 1 μM of test compounds for 2 hr. Transporter activity was then assayed in one-third of the disks immediately after exposure and in the others after 24 hr and 48 hr of washing in fresh FSW (50 mL). FSW was changed after 24 hr. During exposure and washing, dishes were gently rocked at 15°C in the dark. The transporter activity assay was performed with 1 μM rhodamine B as described above.

### Data analysis.

Transport activities in treated gill disks were normalized relative to nontreated controls that were run for each individual. The ratios, which indicate relative increases in fluorescence, were used as a measure of inhibition by each compound at the specified concentrations. The potencies of compounds for inhibiting MXR transporter activity are calculated as concentrations causing 10% and 50% of the maximal inhibition observed for a compound (IC_10_ and IC_50_). IC_10_ and IC_50_ values were determined by probit analysis using a macro program for Microsoft Excel written by J. Greve (Fraunhofer-Institut für Umweltchemie und Ökotoxikologie, Schmallenberg, Germany).

All experiments were performed at least three times using different individuals for each experiment, and mean values and SDs were determined. Statistical analyses were performed using Student’s *t*-test. For correlation analyses, we determined Pearson correlation coefficients. Statistical analyses were conducted using JMP software, version 4.0.4 (SAS Institute Inc., Cary, NC).

## Results

### Method evaluation.

#### Biopsy punch and extraction procedure.

The biopsy punch assay allowed processing large numbers of samples in a standardized way to examine effects of test compounds on efflux transporter activity in gill tissue of mussels. After exposure to rhodamine B and test compounds, the dye was extracted from the biopsy tissue disks in a defined volume of solvent, and the amount of dye was then measured fluorometrically. This approach gave results comparable with the epifluorescence microscopy procedure of measuring accumulation of fluorescent dye in living mussel gills (Cornwall et al. 1995; Eufemia and Epel 1998; Galgani et al. 1996) but was less susceptible to subjective errors connected with the microscopic assay, such as the selection of a tissue area to be measured.

#### Sample homogeneity.

The use of biopsy punches allowed us to obtain samples of equally sized tissue disks from gills from one individual. SDs of dry weight, protein content, and the amount of MXR transporter P-gp (detected with antibody C219 using Western blot techniques) varied by < 10% of the average values in disks from the same animal (data not shown). Furthermore, we found that transporter activity, indicated by the amount of rhodamine B accumulated by the tissue, was consistent among tissue disks from the same individual (e.g., the SD from 18 disks from one individual incubated in FSW with 1 μM rhodamine B was ± 6.5%).

#### Individual variation in transporter activity.

There were considerable differences in basal efflux transporter activity between individual animals. In control tissues, low basal efflux transporter activity is indicated by high accumulation of rhodamine B, whereas high basal efflux activity is indicated by low levels of rhodamine fluorescence (because more dye is effluxed from the cells). We found that these basal rhodamine fluorescence levels varied up to 3.3-fold between individuals. A significant correlation was found between transporter activity and mussel size (quantified by valve length; *p* = 0.003, *n* = 27), but there was no relationship to sex (*p* = 0.32, *n* = 27).

**Figure 1:**
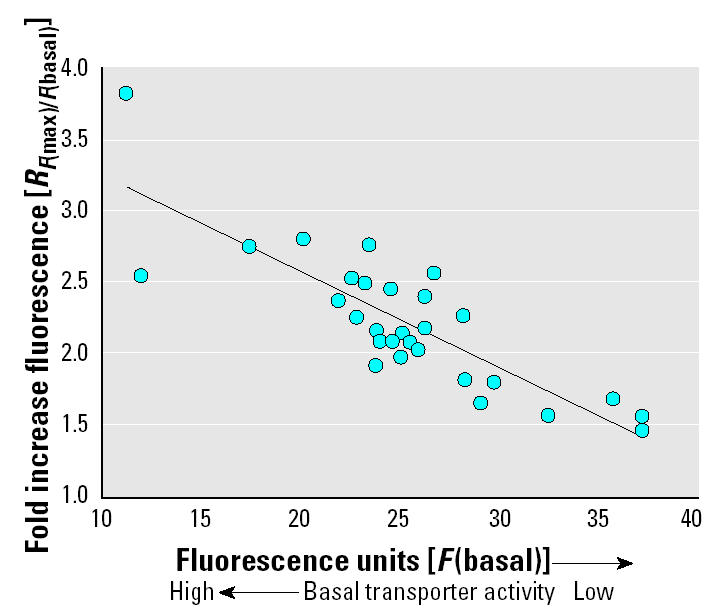
Correlation between basal transporter activity [*F*(basal)] and relative increase in fluorescence in mussel gill tissue with transporter activity inhibited maximally versus basal transporter activity [*R_F_*_(max)/_*_F_*_(basal)_]. *y* = −0.07*x* + 3.93.

These differences in basal activity also correlated with increased uptake of rhodamine (and correspondingly rhodamine fluorescence) when transporter activity was maximally repressed by an inhibitory compound. Using a ratio of maximal fluorescence to basal fluorescence [*R_F_*_(max)/_*_F_*_(basal)_] to compare basal and repressed transporter activity, individuals with low basal transporter activity exhibited a low *R_F_*_(max)/_*_F_*_(basal)_, whereas animals with high basal activity had a high ratio. For example, in a low-activity individual with 37.3 fluorescence units in its control, the *R_F_*_(max)/_*_F_*_(basal)_ was 1.5, whereas in a high-activity individual with 11.3 fluorescence units in the control, the *R_F_*_(max)/_*_F_*_(basal)_ was 3.8. A strong positive correlation (*p* = 6.2 × 10^−8^; *r*^2^ = 0.7; *n* = 27) was found for this relationship (Figure 1).

#### Physiologic indices and long-term exposure studies.

Experiments in which dissected gill tissue was kept in FSW for 48 hr necessitated an assessment of physiology over this period. Oxygen consumption rates measured in isolated gill tissue kept in FSW at 15°C were stable over a period of at least 3 days. Ciliary movement in gills was similarly stable for 3 days (indeed, ciliary movement was unaffected after 7 days of incubation). There were also no evident signs of morphologic deterioration in the tissue. Efflux transporter activity tended to slightly increase over time, as indicated by the lower amounts of rhodamine B in control tissues. After 24 hr, fluorescence values were lower by an average of 8%; after 48 hr they were 13% lower than initial values. A control was therefore run at each time point, and activity was normalized relative to the control tissue.

### Inhibitory effects by test compounds, dose–effect relationships.

**Figure 2:**
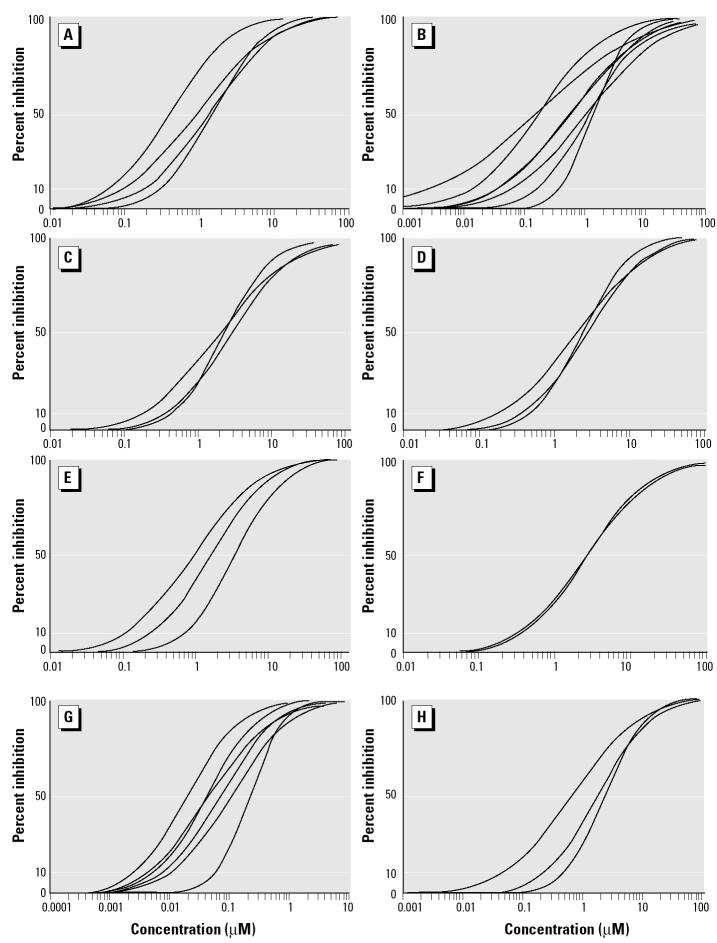
Dose–effect curves for *in vivo* inhibition of MXR transporters in mussel gill tissue by nitromusks [MX (*A*) and MK (*B*)], polycyclic musks [HHCB (*C*), ADBI (*D*), AHTN (*E*), and ATII (*F*)], and reference inhibitors [verapamil (*G*) and quinidine (*H*)]. See “Materials and Methods” for details. Each curve represents data (obtained by probit regression) from an individual animal.

Musks and the P-gp inhibitor reference compounds quinidine and verapamil caused a dose-dependent increase in accumulation of rhodamine B in mussel gill tissue, indicating an inhibition of efflux transporter activity. IC_10_ and IC_50_ values were determined from the dose–effect curves (range of tested concentrations, 0.01–100 μM for musk compounds and quinidine, 0.001–10 μM for verapamil; using 6–11 concentrations per series, including controls). In most cases, curve fits (*r* ^2^) of the probit regressions were between 0.7 and 0.9. Musk concentrations above 5–10 μM did not increase rhodamine B fluorescence, indicating maximum inhibition of the transporter activity at these levels (Figure 2A–F).

The IC_10_ values, used as indicators for low-effect concentrations, showed clear effects in the 0.1–0.4 μM concentration range for musks and quinidine and at 0.01 μM for verapamil (Figure 2, Table 2). The range of IC_50_ values, which were used as a measure for the inhibitory potencies of the test compounds, was 0.7–2.6 μM for the musks and quinidine and 0.08 μM for verapamil (Figure 2, Table 2).

The nitromusks were more effective inhibitors than were the polycyclic musks. When combining the values for the two musk groups, the IC_50_ values for the nitromusks (IC_50_ = 0.82 ± 0.53 μM, *n* = 11) were significantly lower than for the polycyclic musks (IC_50_ = 2.34 ± 0.82 μM, *n* = 12; *p* = 0.0001, Student’s *t*-test).

### Combinatory effects.

Synthetic musks are usually used in combinations in fragrances, and environmental samples often contain several different musk compounds. We therefore tested whether combinations of musks act in a synergistic, antagonistic, or additive fashion in inhibiting transporter activity. We tested treatments containing *a*) a nitromusk mixture (MX and MK), *b*) a polycyclic musk mixture (HHCB and ADBI), and *c*) a nitromusk/polycyclic musk mixture (MX and HHCB). For these experiments, each compound was applied at 0.5 μM to achieve a final total concentration of 1 μM musks. An additional experiment was run with a mixture of two nitromusks (MX and MK) and two polycyclics (HHCB and ADBI), with each compound at 0.25 μM. All compounds were also tested separately at 1 μM.

**Figure 3:**
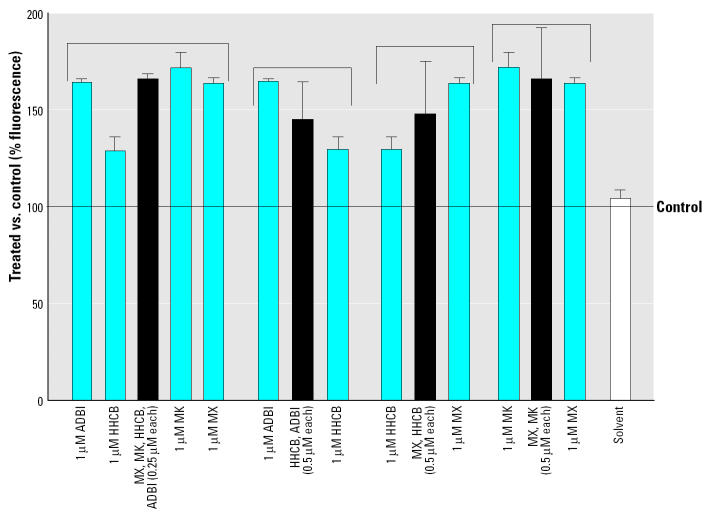
Inhibitory effects by musks as single compounds and in combinations as measured by retention of rhodamine B (see “Materials and Methods” for details). Bars represent percent increase (mean ± SD) of fluorescence versus respective controls; *n* = 7 for experiments with solvent, 1 μM MX, 1 μM MK, 1 μM HHCB, and 1 μM ADBI; *n* = 3 for MX + HHCB, MX + MK, HHCB + ADBI, and MX + MK + HHCB + ADBI. Brackets indicate experiments that are compared.

In all cases, inhibition of transporter activity by mixtures of different musk compounds was intermediate to treatments with single compounds (Figure 3). This indicates that the inhibitory effects of the musks in the mixtures were additive.

### Long-term inhibitory effects.

**Figure 4:**
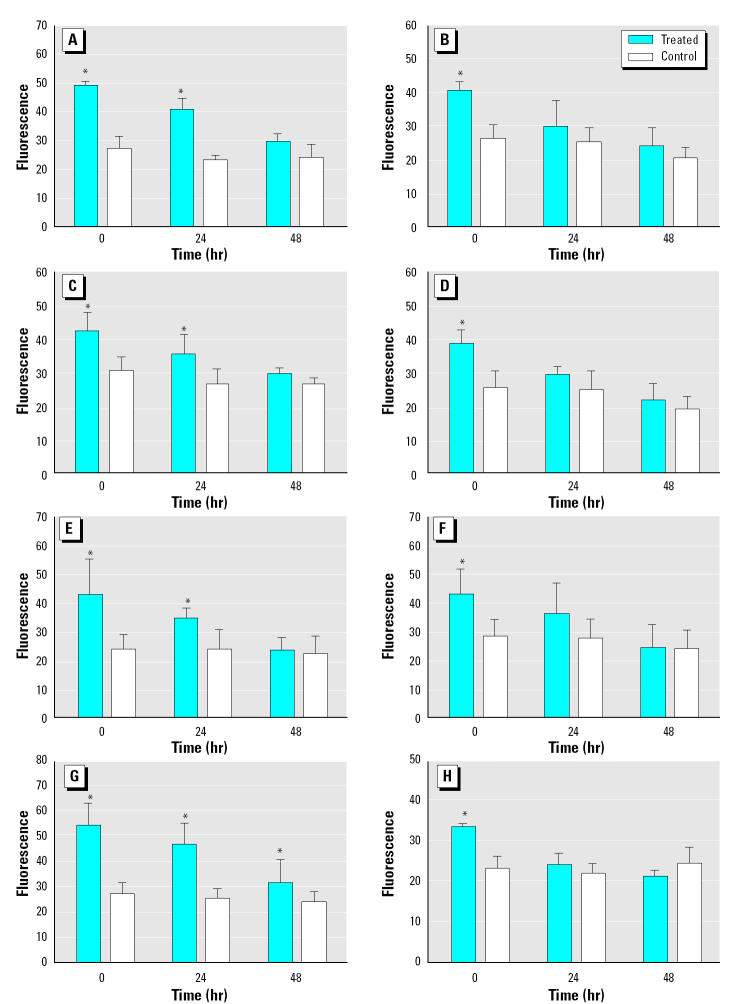
Long-term inhibition of efflux transporters by nitromusks [MX (*A*) and MK (*B*)], polycyclic musks [HHCB (*C*), ADBI (*D*), AHTN (*E*), and ATII (*F*)], and reference inhibitors [verapamil (*G*) and quinidine (*H*)]. Bars represent fluorescence versus respective controls (mean % ± SD); *n* = 3 (11 for verapamil).
*p < 0.05 by paired *t*-test.

We exposed mussel gill disks to test compounds for 2 hr and measured rhodamine uptake directly after the exposure (0 hr) and after 24 and 48 hr of washing the tissue in FSW. Immediately after exposure (0 hr) we incubated a sample of the disks in rhodamine B for 90 min; we found rhodamine uptake to be 38–84% higher in musk treatments compared with respective controls because of transporter inhibition (Figure 4A–F). After 24 hr of washing, another sample of disks was similarly incubated in rhodamine, and levels in the tissues previously exposed to the musks were still 30–74% higher than the controls, indicating continuing inhibition of transporter activity. The differences between treatments versus controls were significant for MX, HHCB, and ATII (*p* < 0.05, paired *t*-test), and there were clear trends of increased rhodamine levels also for MK, ADBI, and AHTN, indicating an inhibitor-related effect after 24 hr (Figure 4). A similar measurement of transport activity at 48 hr showed that activity had now reached the control levels for ATII and AHTN, indicating complete recovery from the inhibitory action of the musks. However, there was still a trend of reduced activity (i.e., higher fluorescence) for the tissues previously incubated in MX, MK, HHCB, and ADBI. The higher fluorescence was consistently seen with these four compounds, indicating an inhibitory effect of these musks even after this time period (Figure 4).

Verapamil, a P-gp reference inhibitor, exhibited strong long-term inhibition, indicated by rhodamine tissue levels that were 88 and 35% higher than the controls at 24 and 48 hr, respectively. In contrast, a different P-gp reference inhibitor, quinidine, showed no long-term effects, with almost complete reversal of inhibition seen after 24 hr of washing (Figure 4).

## Discussion

The present study shows that nitromusk and polycyclic musk compounds inhibit the activity of efflux transporters in the marine mussel *Mytilus californianus* and that these inhibitory effects last for 24–48 hr after termination of exposure to the musks. As part of this work, we also describe an improved efflux transporter assay that allows processing large numbers of tissue samples from one individual and quantifying the potency of chemicals to inhibit MXR transporter activity.

### MXR efflux inhibition by musks.

Both nitromusks and polycyclic musks inhibited efflux transporter activity, with the nitromusks being the more effective inhibitors. The tested musks inhibited transporter activity at a concentration range similar to that for quinidine but were an order of magnitude less effective than verapamil (Figure 2, Table 2). Furthermore, the inhibitory effects of combinations of musks were additive (Figure 3).

Individual mussels differed in basal transporter activity and in sensitivity to treatment with test agents. Differences in size and age may partly explain the variation in activity levels, but the variability is likely to reflect the heterogeneity of the natural population. Thus, stress response levels may be determined by genetic variation and site-specific conditions, such as time of water immersion during tides, wave exposure, temperature, and so forth (Roberts et al. 1997).

**Figure 5:**
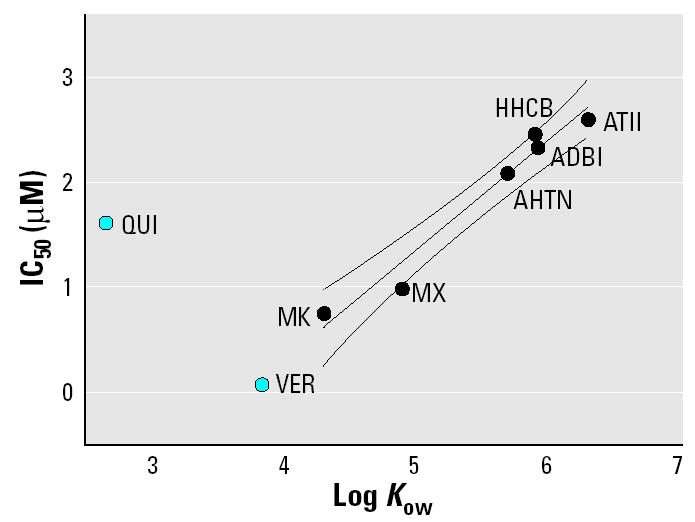
Correlation of MXR inhibitory efficiency (indicated by IC_50_ values) and log *K*_ow_ of test compounds. Abbreviations: QUI, quinidine; VER, verapamil. For the calculation of the regression curve, (with 95% confidence intervals) only musk compounds were included.

Despite this variation among test animals, differences between the different musk species in blocking rhodamine efflux through MXR transporters were clearly visible. These differences in inhibitory capacity most likely result from differences in affinity to substrate binding sites of the transporter, correlated with differences in *K*_ow_ (octanol–water coefficient). Bain and LeBlanc (1996) found that the inhibitory efficiency of a chemical against human P-gp activity was highest for moderately hydrophobic compounds with a log *K*_ow_ in the range of 3.6–4.5. This is consistent with our data showing that the nitromusks, with log *K*_ow_ values of 4.3 and 4.9, were more efficient inhibitors than the more hydrophobic polycyclic musk compounds, with log *K*_ow_ values 5.6–6.3 (Table 1). In fact, IC_50_ values for the musk compounds were strongly correlated with respective log *K*_ow_ values (*p* = 0.0006, *r*^2^ = 0.9, *n* = 6; Figure 5), consistent with the lower inhibitory potency of compounds with a higher hydrophobicity. Verapamil, which was the most effective P-gp inhibitor in our study, also fits the regression line calculated for the musks. Quinidine, however, was less effective as an inhibitor and did not fit this regression line; consistent with its *K*_ow_, which is below the *K*_ow_ range for high-affinity P-gp substrates (Figure 5).

The sensitivity of rhodamine efflux to verapamil, quinidine, and other known P-gp inhibitors/substrates indicates that the MXR phenomenon in *Mytilus* is associated with a P-gp–like efflux transporter (Cornwall et al. 1995; Galgani et al. 1996; Minier et al. 1993; Smital et al. 2003). Other transporters, such as an MRP (Lüdeking and Köhler 2002), may be present, but the substrate and inhibitor profile suggests a dominant activity of a P-gp type transporter, which is most likely the target of the musk compounds.

### Long-term inhibitory effects of musks.

Inhibitory effects of musk compounds and verapamil were still present 24–48 hr after removal of the inhibitors (Figure 4). To our knowledge, this is the first demonstration of long-term inhibitory effects of MXR modulators. These results were unexpected because efflux transporters are involved in removal of xenobiotics from cells; therefore, quicker recovery of the transporter activity would have been anticipated.

The inhibition indicates that the musks and verapamil remain accessible to the efflux transporters for 24–48 hr after removal of the compounds from the medium. This could be related to the hydrophobicity of the compounds and/or their affinity for the transporters. Hydrophobic compounds will accumulate in the cell membrane during exposure and could affect efflux transporters indirectly through membrane effects (Ferté 2000), or the membranes could directly serve as reservoirs for slow release of the chemicals, which could then bind to the active sites of the transporter proteins. Alternatively, the chemicals might have a high affinity for specific sites of the transporters such as substrate binding sites or other functional sites (e.g., ATP-binding site) from where they are only slowly released, with resultant long-term inhibition.

### Potential environmental relevance of synthetic musks as chemosensitizers.

It was first suggested by Kurelec (1997) that environmental pollutants may act as chemosensitizers by compromising the MXR system, allowing other toxicants, which would normally be excluded by the MXR transporters, to enter the cell. Kurelec pointed out that chemosensitizers could include chemicals of low toxicity, which would present an unanticipated environmental or human health risk. A broad range of anthropogenic chemicals such as pesticides, pharmaceuticals, and some polyaromatic hydrocarbons have been found to inhibit MXR transporters by blocking efflux of fluorescent or radio-labeled substrates in human cells or cells of aquatic organisms (Bain and LeBlanc 1996; Britvic and Kurelec 1999; Cornwall et al. 1995; Eufemia and Epel 1998; Kurelec 1992; Smital and Kurelec 1998b; Toomey and Epel 1993). When water samples from differently contaminated field sites were tested for their inhibitory potential for MXR transporters, greater inhibition by samples containing high levels of anthropogenic pollutants was detected (Smital and Kurelec 1997). This suggests that levels of anthropogenic pollutants currently found in the environment have the potential to interfere with normal animal defense mechanisms. Recent extensions of this work have shown that these MXR chemosensitizers increased the potency of toxic or mutagenic transporter substrates (Britvic and Kurelec 1999; Smital and Kurelec 1998a).

Our data indicate that synthetic musks are also chemosensitizers and could therefore have indirect effects by allowing normally excluded toxicants to permeate cells. Earlier studies have pointed to other indirect toxic effects of nitromusks. For example, MK and MX both induce P450-dependent oxygenases (Lehman-McKeeman et al. 1995; Mersch-Sundermann et al. 1996, 2001; Suter-Eichenberger et al. 1999) and therefore could cause increased transformation of other environmental chemicals that are mutagenic in their transformed form such as benzo(*a*)pyrene, 2-aminoanthracene, and aflatoxin B1 (Mersch-Sundermann et al. 1996, 2001).

Do the effects of synthetic musks as MXR inhibitors apply to real-world situations? The concentration range in which musks are effective inhibitors [10^−6^–10^−7^ M (ppb) range] is several orders of magnitude higher than concentrations reported for water samples from the environment [10^−9^–10^−12^ M (ppt) range] (Rimkus 1999). However, musks are concentrated in sediments (Winkler et al. 1998), which could make them available to bottom dwellers. More important, their hydrophobicity results in bioaccumulation and levels in tissues of aquatic organisms are 10^1^- to 10^4^-fold higher, and in the lipid fraction may be > 10^5^-fold above environmental levels (Gatermann et al. 2002; Rimkus 1999). Association with lipids may be especially relevant to MXR transporters, which reside in the cell membrane and hence are more directly exposed to fat-soluble compounds such as the musks. These considerations indicate that even if ambient concentrations are low, long-term exposure will lead to tissue burdens that could inhibit MXR function indirectly via membrane effects or directly via high-affinity binding to transporter sites.

The unexpected long-term effects of the musks are troubling. One consequence of such long-term inhibition of transporters is that short-term events could have continuing consequences. Thus, the effects of short-term incidents such as storm-water runoffs or chemical spills could continue after such events if there is accumulation of chemosensitizers in the tissue, or prolonged inhibition as a result of the acute exposure.

Musks are typically present in the environment with other contaminants (Fromme et al. 2001; Kallenborn et al. 1999) that could well include other transporter modulators. As shown, the effects of combinations of P-gp inhibitors are additive, and therefore musks could be part of a suite of pollutants that contribute to chemosensitizing effects in nature. In fact, as first suggested by Daughton and Ternes (1999), this could be an unanticipated consequence of chemicals such as the PPCPs. Additive effects of low concentrations of many compounds could affect the MXR transporters, and this would be magnified by the sheer numbers of different components of the PPCPs.

### Musks and other environmental chemosensitizers—a human health risk?

This work on the musks, although focused on aquatic organisms, also points to unsuspected effects of these chemicals on human health. MXR efflux pumps are widely distributed in mammalian tissues, where they are a crucial part of the cellular defense against cytotoxins (Cordon-Cardo et al. 1990). The effects of environmental contaminants as chemosensitizers have not been studied in humans; however, inhibitors of efflux transporters used in cancer therapy can lead to increased permeability of healthy tissues to transporter substrates (Luker et al. 1997), indicating that chemosensitizers could pose a health threat.

Because synthetic musks are present in human tissue samples, the question arises of whether they could also be relevant as chemosensitizers in humans. Concentrations of MX found in body fat and breast milk are in the range of 0.2–0.3 μmol/kg, and are in the nanomolar range in blood plasma (Käfferlein et al. 1998). These values represent body burdens that are one to two orders of magnitude below the effective concentrations seen in the present study on mussel tissue; however, they could contribute to additive effects with other compounds.

The present study especially points to the need to screen musks and other environmental chemicals that accumulate in humans to determine if they are also chemosensitizers of MXR-related transporters. It will be especially critical to ascertain whether they cause long-term effects similar to those seen in our study. Effects on efflux systems could result in unanticipated accumulation of toxicants in humans and confound safety predictions of seemingly innocuous chemicals.

## Figures and Tables

**Table 1:** Names, CAS numbers, formulas, structures, molecular weights, and log *K*_ow_ values for artificial musks and MXR model substrates and inhibitors.

Chemical and trade names	CAS No.	Formula	Structure	Molecular weight	Log *K*_ow_
Musk xylene (MX) 1-*tert*-Butyl-3,5-dimethyl-2,4,6-trinitrobenzene	81–15–2	C_12_H_15_N_3_O_6_	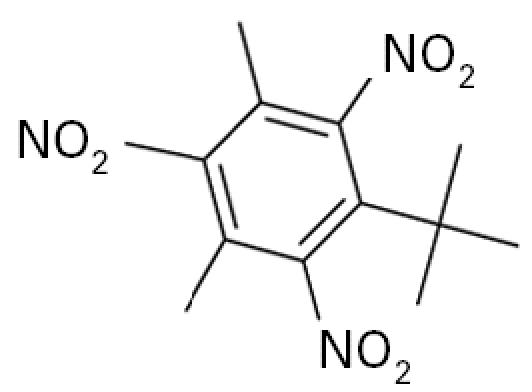	297.3	4.9*^a^*
Musk ketone (MK) 1-*tert*-Butyl-3,5-dimethyl-2,6-dinitro-4-acetylbenzene	81–14–1	C_14_H_18_N_2_O_5_	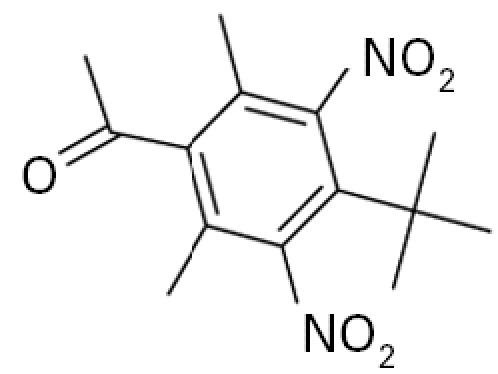	294.3	4.3*^a^*
Galaxolide (HHCB) 1,3,4,6,7,8-Hexahydro-4,6,6,7,8,8-hexamethyl-cyclopenta-γ-[2]-benzopyran	1222–05–5	C_18_H_26_O	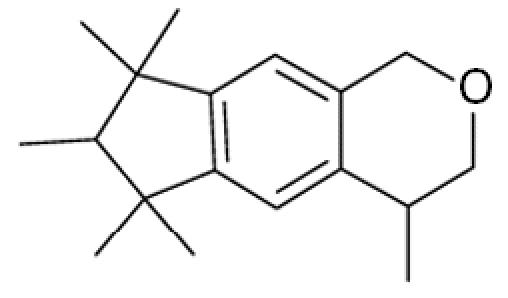	258.4	5.9*^a^*
Celestolide, Crysolide (ADBI) 4-Acetyl-1,1-dimethyl-6-*tert*-butylindane	13171–00–1	C_17_H_24_O	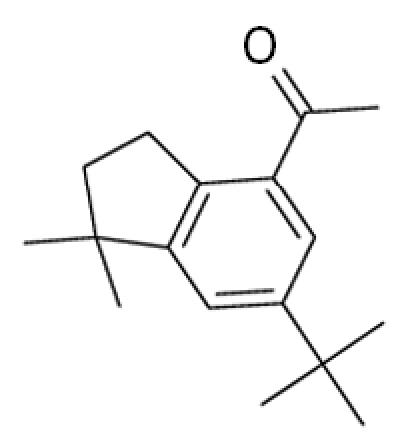	244.4	5.9*^a^*
Tonalide, Tetralide, Fixolide (AHTN) 7-Acetyl-1,1,3,4,4,6-hexamethyl-tetrahydronaphthalene	21145–77–7	C_18_H_26_O	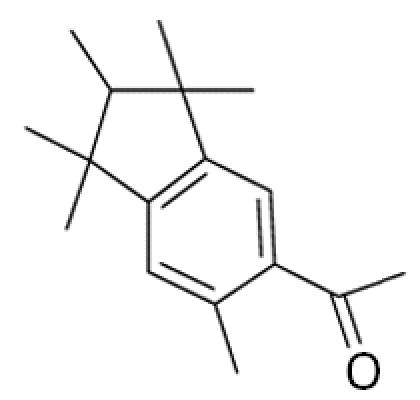	244.4	5.7*^a^*
Traseolide (ATII) 5-Acetyl-1,1,2,6-tetramethyl-3-isopropylindane	68140–48–7	C_18_H_26_O	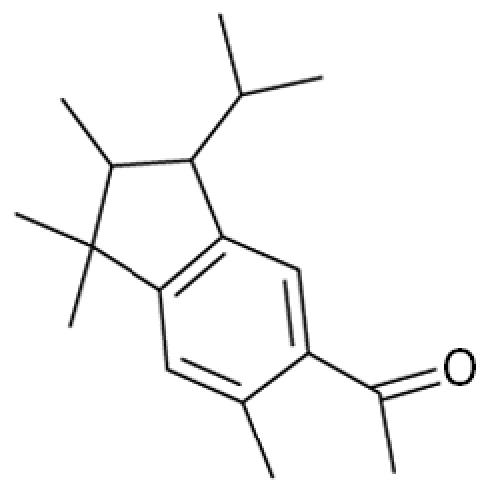	258.4	6.3*^a^*
Quinidine	56–54–2	C_20_H_24_N_2_O_2_	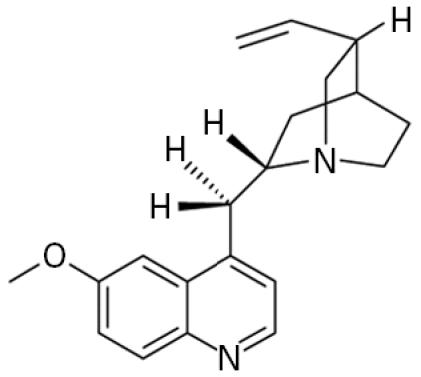	324.4	2.8*^b^*
Verapamil	52–53–9	C_27_H_38_N_2_O_4_	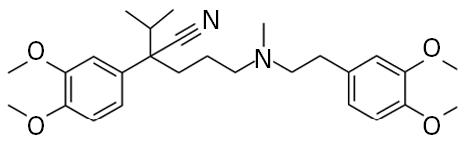	454.6	4.5*^b^*
Rhodamine B	81–88–9	C_28_H_31_ClN_2_O_3_	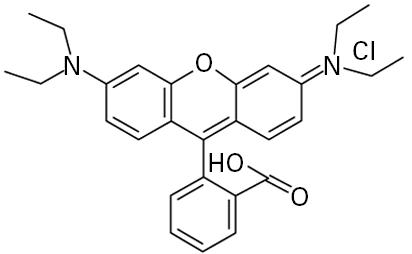	479.0	1.5*^c^*

a Data from Balk et al. (2001).

b Data from Wang et al. (2003).

c Data from Liu (2004).

**Table 2:** IC_10_ and IC_50_ values (mean ± SD) of nitromusks, polycyclic musks, and reference inhibitors verapamil and quinidine for efflux transporters in mussel gills.

	IC_10_	IC_50_	*n*
Nitromusks			
MX	0.14 ± 0.12	0.97 ± 0.63	4
MK	0.09 ± 0.12	0.74 ± 0.49	7
Polycyclic musks			
HHCB	0.37 ± 0.16	2.43 ± 0.45	3
ADBI	0.39 ± 0.34	2.32 ± 1.18	3
AHTN	0.35 ± 0.31	2.05 ± 0.31	3
ATII	0.32 ± 0.04	2.56 ± 0.07	3
Verapamil	0.01 ± 0.02	0.08 ± 0.07	6
Quinidine	0.24 ± 0.20	1.59 ± 0.85	3

Data were obtained by probit regression.

## References

[b1-ehp0113-000017] Api AM, Smith RL, Pipino S, Marczylo T, De Matteis F (2004). Evaluation of the oral subchronic toxicity of AHTN (7-acetyl-1,1,3,4,4,6-hexamethyl-1,2,3,4-tetrahydronaphthalene) in the rat. Food Chem Toxicol.

[b2-ehp0113-000017] Bain LJ, LeBlanc GA (1996). Interaction of structurally diverse pesticides with the human MDR1 gene product P-glycoprotein. Toxicol Appl Pharmacol.

[b3-ehp0113-000017] Bain LJ, McLachlan JB, LeBlanc GA (1997). Structure–activity relationships for xenobiotic transport substrates and inhibitory ligands of P-glycoprotein. Environ Health Perspect.

[b4-ehp0113-000017] Balk F Blok H Salvito D 2001. Environmental risks of musk fragrance ingredients. In: Pharmaceuticals and Personal Care Products in the Environment—Scientific and Regulatory Issues (Daughton CG, Jones-Lepp TL, eds). Washington, DC:American Chemical Society, 168–190.

[b5-ehp0113-000017] Balk F, Ford RA (1999a). Environmental risk assessment for the polycyclic musks AHTN and HHCB in the EU. I. Fate and exposure assessment. Toxicol Lett.

[b6-ehp0113-000017] Balk F, Ford RA (1999b). Environmental risk assessment for the polycyclic musks, AHTN and HHCB. II. Effect assessment and risk characterisation. Toxicol Lett.

[b7-ehp0113-000017] Behechti A, Schramm KW, Attar A, Niederfellner J, Kettrup A (1998). Acute aquatic toxicities of four musk xylene derivatives on *Daphnia magna*. Water Res.

[b8-ehp0113-000017] Bester K, Hühnerfuss H, Lange W, Rimkus GG, Theobald N (1998). Results of non target screening of lipophilic organic pollutants in the German Bight II: polycyclic musk fragrances. Water Res.

[b9-ehp0113-000017] Bitsch N, Dudas C, Körner W, Failing K, Biselli S, Rimkus G (2002). Estrogenic activity of musk fragrances detected by the E-screen assay using human MCF-7 cells. Arch Environ Contam Toxicol.

[b10-ehp0113-000017] Breitholtz M, Wollenberger L, Dinan L (2003). Effects of four synthetic musks on the life cycle of the harpacticoid copepod *Nitocra spinipes*. Aquat Toxicol.

[b11-ehp0113-000017] Britvic S, Kurelec B (1999). The effect of inhibitors of multixenobiotic resistance mechanism on the production of mutagens by *Dreissena polymorpha* in waters spiked with premutagens. Aquat Toxicol.

[b12-ehp0113-000017] Chou YJ, Dietrich DR (1999a). Interactions of nitromusk parent compounds and their amino-metabolites with the estrogen receptors of rainbow trout (*Oncorhynchus mykiss*) and the South African clawed frog (*Xenopus laevis*). Toxicol Lett.

[b13-ehp0113-000017] Chou YJ, Dietrich DR (1999b). Toxicity of nitromusks in early lifestages of South African clawed frog (*Xenopus laevis*) and zebrafish (*Danio rerio*). Toxicol Lett.

[b14-ehp0113-000017] Cordon-Cardo C, O’Brien JP, Boccia J, Casals D, Bertino JR, Melamed MR (1990). Expression of the multidrug resistance gene product (P-glycoprotein) in human normal and tumor tissues. J Histochem Cytochem.

[b15-ehp0113-000017] Cornwall R, Toomey BH, Bard S, Bacon C, Jarman WM, Epel D (1995). Characterization of multixenobiotic/multidrug transport in the gills of the mussel *Mytilus californianus* and identification of environmental substrates. Aquat Toxicol.

[b16-ehp0113-000017] Daughton CG, Ternes TA (1999). Pharmaceuticals and personal care products in the environment: agents of subtle change?. Environ Health Perspect.

[b17-ehp0113-000017] Draisci R, Marchiafava C, Ferretti E, Palleschi L, Catellani G, Anastasio A (1998). Evaluation of musk contamination of freshwater fish in Italy by accelerated solvent extraction and gas chromatography with mass spectrometric detection. J Chromatogr A.

[b18-ehp0113-000017] Epel D (1998). Use of multidrug transporters as first lines of defense against toxins in aquatic organisms. Comp Biochem Physiol A.

[b19-ehp0113-000017] Epel D Smital T 2001. Multidrug-multixenobiotic transporters and their significance with respect to environmental levels of pharmaceuticals and personal care products. In: Pharmaceuticals and Personal Care Products in the Environment—Scientific and Regulatory Issues (Daughton CG, Jones-Lepp TL, eds). Washington, DC:American Chemical Society, 244–263.

[b20-ehp0113-000017] Eufemia NA, Epel D (1998). The multixenobiotic defense mechanism in mussels is induced by substrates and non-substrates: implications for a general stress response. Mar Environ Res.

[b21-ehp0113-000017] Ferté J (2000). Analysis of the tangled relationships between P-glycoprotein-mediated multidrug resistance and the lipid phase of the cell membrane. Eur J Biochem.

[b22-ehp0113-000017] Fromme H, Otto T, Pilz K (2001). Polycyclic musk fragrances in different environmental compartments in Berlin (Germany). Water Res.

[b23-ehp0113-000017] Frosch PJ, Pilz B, Andersen KE, Burrows D, Camarasa JG, Dooms-Goossens A (1995). Patch testing with fragrances: results of a multicenter study of the European Environmental and Contact Dermatitis Research Group with 48 frequently used constituents of perfumes. Contact Dermatitis.

[b24-ehp0113-000017] Galgani F, Cornwall R, Toomey BH, Epel DD (1996). Interaction of environmental xenobiotics with a multixenobiotic defense mechanism in the bay mussel *Mytilus galloprovincialis* from the coast of California. Environ Toxicol Chem.

[b25-ehp0113-000017] Gatermann R, Biselli S, Hühnerfuss H, Rimkus GG, Hecker M, Karbe L (2002). Synthetic musks in the environment. Part 1: Species-dependent bioaccumulation of polycyclic and nitro musk fragrances in freshwater fish and mussels. Arch Environ Contam Toxicol.

[b26-ehp0113-000017] Käfferlein HU, Angerer J (2001). Trends in the musk xylene concentrations in plasma samples from the general population from 1992/1993 to 1998 and the relevance of dermal uptake. Int Arch Occup Environ Health.

[b27-ehp0113-000017] Käfferlein HU, Goen T, Angerer J (1998). Musk xylene: analysis, occurrence, kinetics, and toxicology. Crit Rev Toxicol.

[b28-ehp0113-000017] Kallenborn R, Gatermann R, Rimkus GG (1999). Synthetic musks in environmental samples: indicator compounds with relevant properties for environmental monitoring. J Environ Monit.

[b29-ehp0113-000017] Kester M, Bulduk S, Tibboel D, Meinl W, Glatt H, Falany C (2000). Potent inhibition of estrogen sulfotransferase by hydroxylated PCB metabolites: a novel pathway explaining the estrogenic activity of PCBs. Endocrinology.

[b30-ehp0113-000017] Kupper T, Berset JD, Etter-Holzer R, Furrer R, Tarradellas J (2004). Concentrations and specific loads of polycyclic musks in sewage sludge originating from a monitoring network in Switzerland. Chemosphere.

[b31-ehp0113-000017] Kurelec B (1992). The multixenobiotic resistance mechanism in aquatic organisms. Crit Rev Toxicol.

[b32-ehp0113-000017] Kurelec B (1995). Reversion of the multixenobiotic resistance mechanism in gills of marine mussel *Mytilus galloprovincialis* by a model inhibitor and environmental modulators of P170-glycoprotein. Aquat Toxicol.

[b33-ehp0113-000017] Kurelec B (1997). A new type of hazardous chemical: the chemosensitizers of multixenobiotic resistance. Environ Health Perspect.

[b34-ehp0113-000017] Lehman-McKeeman LD, Caudill D, Young JA, Dierckman TA (1995). Musk xylene induces and inhibits mouse hepatic cytochrome P-450 2B enzymes. Biochem Biophys Res Commun.

[b35-ehp0113-000017] Liebl B, Ehrenstorfer S (1993). Nitromoschusverbindungen in der Frauenmilch [in German]. Gesundheitswesen.

[b36-ehp0113-000017] Liu Z (2004). Confocal laser scanning microscopy—an attractive tool for studying the uptake of xenobiotics into plant foliage. J Microsc.

[b37-ehp0113-000017] Luckenbach T, Corsi I, Epel D (2004). Fatal attraction: synthetic musk fragrances compromise multixenobiotic defense systems in mussels. Mar Environ Res.

[b38-ehp0113-000017] Lüdeking A, Köhler A (2002). Identification of six mRNA sequences of genes related to multixenobiotic resistance (MXR) and biotransformation in *Mytilus edulis*. Mar Ecol-Prog Ser.

[b39-ehp0113-000017] Luker GD, Fracasso PM, Dobkin J, Piwnica-Worms D (1997). Modulation of the multidrug resistance P-glycoprotein: detection with technetium-99m-sestamibi in vivo. J Nucl Med.

[b40-ehp0113-000017] Mersch-Sundermann V, Emig M, Reinhardt A (1996). Nitro musks are cogenotoxicants by inducing toxifying enzymes in the rat. Mutat Res.

[b41-ehp0113-000017] Mersch-Sundermann V, Schneider H, Freywald C, Jenter C, Parzefall W, Knasmuller S (2001). Musk ketone enhances benzo(*a*)pyrene induced mutagenicity in human derived Hep G2 cells. Mutat Res.

[b42-ehp0113-000017] Minier C, Akcha F, Galgani F (1993). P-glycoprotein expression in *Crassostrea gigas* and *Mytilus edulis* in polluted seawater. Comp Biochem Physiol B.

[b43-ehp0113-000017] Neyfakh AA (1988). Use of fluorescent dyes as molecular probes for the study of multidrug resistance. Exp Cell Res.

[b44-ehp0113-000017] Osemwengi LI, Steinberg S (2001). On-site solid-phase extraction and laboratory analysis of ultra-trace synthetic musks in municipal sewage effluent using gas chromatographymass spectrometry in the full-scan mode. J Chromatogr A.

[b45-ehp0113-000017] Ott M, Failing K, Lang U, Schubring C, Gent HJ, Georgii S (1999). Contamination of human milk in Middle Hesse, Germany—a cross-sectional study on the changing levels of chlorinated pesticides, PCB congeners and recent levels of nitro musks. Chemosphere.

[b46-ehp0113-000017] Paasivirta J, Sinkkonen S, Rantalainen AL, Broman D, Zebuhr Y (2002). Temperature dependent properties of environmentally important synthetic musks. Environ Sci Pollut Res Int.

[b47-ehp0113-000017] Peck AM, Hornbuckle KC (2004). Synthetic musk fragrances in Lake Michigan. Environ Sci Technol.

[b48-ehp0113-000017] Ricking M, Schwarzbauer J, Hellou J, Svenson A, Zitko V (2003). Polycyclic aromatic musk compounds in sewage treatment plant effluents of Canada and Sweden: first results. Mar Pollut Bull.

[b49-ehp0113-000017] Rimkus GG (1999). Polycyclic musk fragrances in the aquatic environment. Toxicol Lett.

[b50-ehp0113-000017] Rimkus GG, Butte W, Geyer HJ (1997). Critical considerations on the analysis and bioaccumulation of musk xylene and other synthetic nitro musks in fish. Chemosphere.

[b51-ehp0113-000017] Rimkus GG, Gatermann R, Hühnerfuss H (1999). Musk xylene and musk ketone amino metabolites in the aquatic environment. Toxicol Lett.

[b52-ehp0113-000017] Rimkus GG, Wolf M (1995). Nitro musk fragrances in biota from freshwater and marine environment. Chemosphere.

[b53-ehp0113-000017] Rimkus GG, Wolf M (1996). Polycyclic musk fragrances in human adipose tissue and human milk. Chemosphere.

[b54-ehp0113-000017] Roberts DA, Hofmann GE, Somero GN (1997). Heat-shock protein expression in *Mytilus californianus*: acclimatization (seasonal and tidal-height comparisons) and acclimation effects. Biol Bull.

[b55-ehp0113-000017] Sanderson J, Letcher R, Heneweer M, Giesy J, van den Berg M (2001). Effects of chloro-*s*-triazine herbicides and metabolites on aromatase activity in various human cell lines and on vitellogenin production in male carp hepatocytes. Environ Health Perspect.

[b56-ehp0113-000017] Schmeiser HH, Gminski R, Mersch-Sundermann V (2001). Evaluation of health risks caused by musk ketone. Int J Hyg Environ Health.

[b57-ehp0113-000017] Smital T, Kurelec B (1997). Inhibitors of the multixenobiotic resistance mechanism in natural waters: in vivo demonstration of their effects. Environ Toxicol Chem.

[b58-ehp0113-000017] Smital T, Kurelec B (1998a). The activity of multixenobiotic resistance mechanism determined by rhodamine B-efflux method as a biomarker of exposure. Mar Environ Res.

[b59-ehp0113-000017] Smital T, Kurelec B (1998b). The chemosensitizers of multixenobiotic resistance mechanism in aquatic invertebrates: a new class of pollutants. Mutat Res.

[b60-ehp0113-000017] Smital T, Luckenbach T, Sauerborn R, Hamdoun AM, Vega RL, Epel D (2004). Emerging contaminants—pesticides, PPCPs, microbial degradation products and natural substances as inhibitors of multixenobiotic defense in aquatic organisms. Mutat Res.

[b61-ehp0113-000017] Smital T, Sauerborn R, Hackenberger BK (2003). Inducibility of the P-glycoprotein transport activity in the marine mussel *Mytilus galloprovincialis* and the freshwater mussel *Dreissena polymorpha*. Aquat Toxicol.

[b62-ehp0113-000017] Suter-Eichenberger R, Boelsterli UA, Conscience-Egli M, Lichtensteiger W, Schlumpf M (1999). CYP 450 enzyme induction by chronic oral musk xylene in adult and developing rats. Toxicol Lett.

[b63-ehp0113-000017] Tas JW Balk F 1997. Environmental Risk Assessment of the Polycyclic Musks AHTN and HHCB According to the EU-TGD. RIVM Report No. 601 503 008. Bilthoven, Netherlands:National Institute of Public Health and the Environment.

[b64-ehp0113-000017] Tas JW, Balk F, Ford RA, van de Plassche EJ (1997). Environmental risk assessment of musk ketone and musk xylene in the Netherlands in accordance with the EU-TGD. Chemosphere.

[b65-ehp0113-000017] Toomey BH, Epel D (1993). Multixenobiotic resistance in *Urechis caupo* embryos: protection from environmental toxins. Biol Bull.

[b66-ehp0113-000017] Wang RB, Kuo CL, Lien LL, Lien EJ (2003). Structure-activity relationship: analyses of P-glycoprotein substrates and inhibitors. J Clin Pharm Ther.

[b67-ehp0113-000017] White KM 1937. Mytilus. Liverpool, UK:University Press of Liverpool.

[b68-ehp0113-000017] Winkler M, Kopf G, Hauptvogel C, Neu T (1998). Fate of artificial musk fragrances associated with suspended particulate matter (SPM) from the River Elbe (Germany) in comparison to other organic contaminants. Chemosphere.

[b69-ehp0113-000017] Wollenberger L, Breitholtz M, Ole Kusk K, Bengtsson BE (2003). Inhibition of larval development of the marine copepod *Acartia tonsa* by four synthetic musk substances. Sci Total Environ.

[b70-ehp0113-000017] Yamagishi T, Miyazaki T, Horii S, Akiyama K (1983). Synthetic musk residues in biota and water from Tama River and Tokyo Bay (Japan). Arch Environ Contam Toxicol.

[b71-ehp0113-000017] Yamagishi T, Miyazaki T, Horii S, Kaneko S (1981). Identification of musk xylene and musk ketone in freshwater fish collected from the Tama River, Tokyo. Bull Environ Contam Toxicol.

